# The Effectiveness of Combined Mirror Therapy and Contralateral Controlled Functional Electrical Stimulation Therapy in a Stroke Patient With Upper Limb Motor Paralysis: A Case Report

**DOI:** 10.7759/cureus.84433

**Published:** 2025-05-19

**Authors:** Keiichiro Aoki, Kengo Uchibori, Takayuki Watabe, Akira Yoshikawa, Nobiyuki Kawate

**Affiliations:** 1 Division of Occupational Therapy, Department of Rehabilitation, School of Nursing and Rehabilitation Sciences, Showa Medical University, Yokohama, JPN; 2 Rehabilitation Center, Showa Medical University Fujigaoka Rehabilitation Hospital, Yokohama, JPN; 3 Division of Health Science Education, School of Nursing and Rehabilitation Sciences, Showa Medical University, Yokohama, JPN; 4 Department of Rehabilitation Medicine, Graduate School of Medicine, Showa Medical University, Tokyo, JPN

**Keywords:** case report, electrical stimulation, mirror therapy, stroke rehabilitation, upper limb paralysis

## Abstract

Severe upper limb motor paralysis following a stroke significantly limits patients' daily function and quality of life. While traditional therapies like constraint-induced therapy, muscle strengthening, and exercise are commonly used, they often lack effectiveness for severe cases due to their reliance on voluntary movement. In contrast, mirror therapy and contralaterally controlled functional electrical stimulation (CCFES) have shown potential by directly engaging neural pathways to promote recovery. This case report describes combining two methods to create a more effective approach for enhancing motor recovery in patients with severe upper limb deficits.

A 59-year-old Japanese male presented with left hemiplegia following a right subcortical hemorrhage. Following craniotomy and cranioplasty, he was transferred to our facility for rehabilitation. Upon initiation of occupational therapy, comprehensive assessments revealed profound motor impairments in the left upper extremity, with a Fugl-Meyer Assessment-Upper Extremity (FMA-UE) score of 17/66 at the start of therapy and initial Motor Activity Log (MAL) scores of amount of use (AOU) 1.5 and quality of movement (QOM) 1.0 at week four. A combined regimen of mirror therapy and CCFES was administered over 16 weeks. During this time, the patient exhibited substantial improvements in motor function, with the FMA-UE score increasing to 52/66 and the MAL scores improving to AOU 3.6 and QOM 3.9 by week 16. This study suggests that a rehabilitation strategy combining mirror therapy with CCFES can effectively enhance motor recovery in patients with severe upper limb motor paralysis. However, these findings should be interpreted with caution as they are based on a preliminary single-patient case study. Further research with larger sample sizes is needed to confirm the efficacy of this combined approach and assess its long-term benefits.

## Introduction

Rehabilitation for severe upper limb motor paralysis following a stroke is very critical, as it significantly affects patients' ability to engage in social activities and impacts their overall quality of life. Traditional therapies, such as constraint-induced therapy, muscle strengthening training, and exercise therapy, are commonly employed during the subacute phase of recovery [1-3]. While these approaches have proven beneficial for patients with mild to moderate impairments, they often fall short when addressing the more complex needs of those with severe upper limb paralysis. For example, constraint-induced therapy relies on a minimum level of voluntary movement that may not be feasible for patients with severe motor deficits, reducing its effectiveness in this population [1]. Similarly, muscle strengthening training and exercise therapy can lack the specific neural activation required to drive significant improvements in cases of extensive motor impairment [2,3].

Mirror therapy and contralaterally controlled functional electrical stimulation (CCFES) have each shown promise in promoting neural reorganization and motor recovery in patients with severe paralysis. Mirror therapy works by creating the illusion of movement in the paralyzed hand through the reflection of the non-paralyzed hand's movements, which activates the primary motor cortex and stimulates the neural networks involved in movement [4]. CCFES enhances this process by using sensors to detect muscle activity in the non-paralyzed limb and delivering corresponding electrical stimulation to the paralyzed muscles, facilitating synchronized bilateral movements and further supporting motor recovery [5].

This case report hypothesizes that the combination of mirror therapy and CCFES would enhance the activation of neural networks responsible for motor function, restore balance between the cerebral hemispheres, and ameliorate motor impairments associated with severe upper limb paralysis. This report utilizes a prospective, single-subject experimental design to closely observe and analyze the response of an individual patient to this innovative treatment approach for severe upper limb motor paralysis. This design was chosen to enable a detailed assessment of the patient’s motor function progression over time and to evaluate the therapeutic impact of the combined intervention.

Although recent randomized controlled trials have explored this combination [6], these studies have primarily focused on short-term motor function improvements, leaving a gap in the systematic evaluation of its long-term efficacy in subacute stroke rehabilitation. Therefore, in this report, we assessed the effectiveness of this combined rehabilitation strategy for severe upper limb motor paralysis by focusing on a case with long-term sustained functional improvements.

## Case presentation

**Figure 1:**
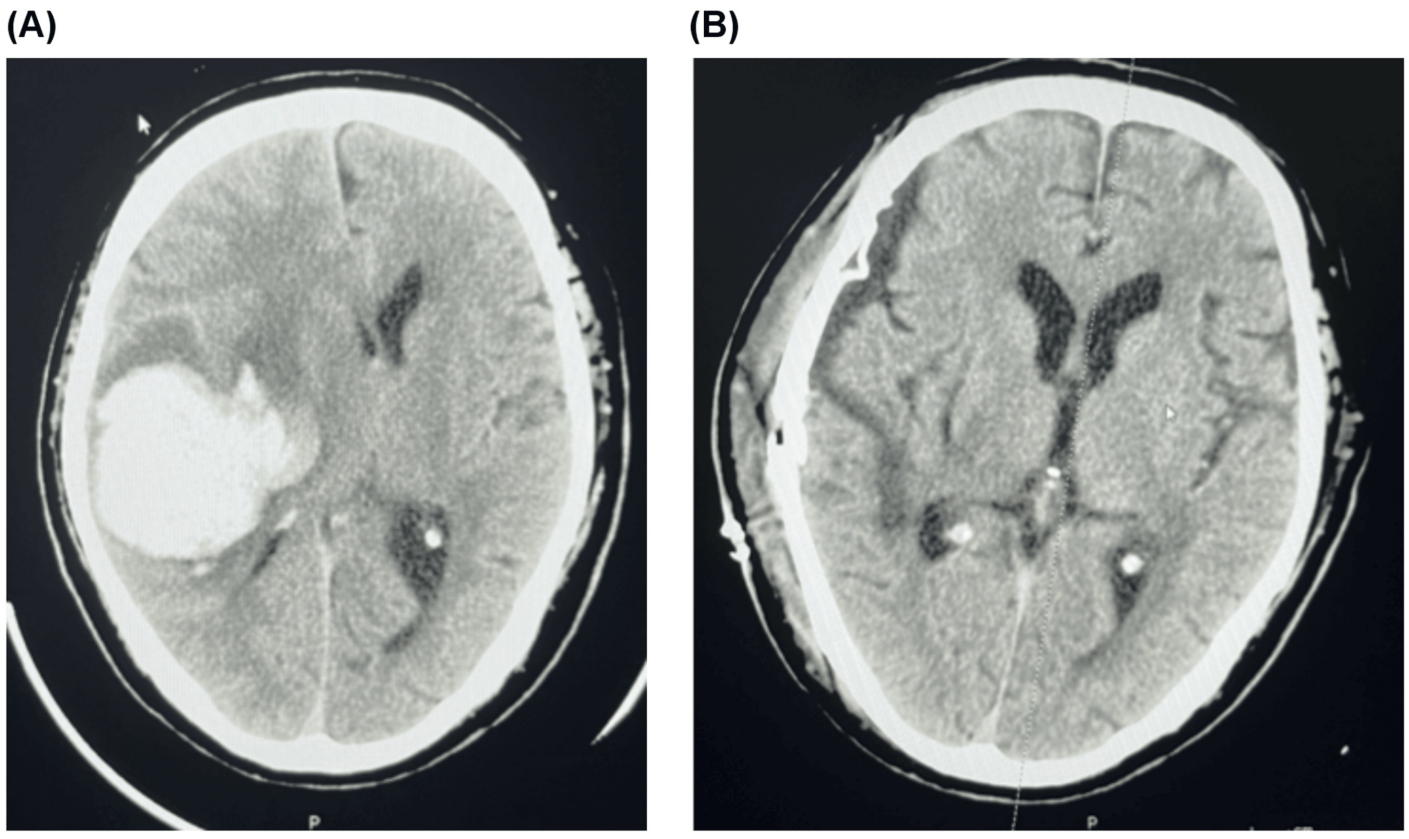
Axial CT images showing the patient’s right subcortical hemorrhage before and after surgical intervention (A) Axial image of preoperative CT scan, taken on day one, showing a right subcortical hemorrhage prior to craniotomy for hematoma evacuation. (B) Axial image of postoperative CT scan, taken after cranioplasty on day 40, showing the surgical results CT: computed tomography

The patient was a 59-year-old Japanese male who developed left hemiplegia following a right subcortical hemorrhage. He was discovered to have collapsed (day one) and was promptly transported to Fujigaoka Rehabilitation Hospital. A CT scan confirmed the presence of a right subcortical hemorrhage, and the patient underwent craniotomy for hematoma evacuation on the same day (Figure 1). Subsequently, on day 40, he underwent cranioplasty. On day 54, he was transferred to our facility for rehabilitation, marking the initiation of the intervention.

At the outset of occupational therapy, several assessments were performed. The Brunnstrom Recovery Stage (BRS) was recorded as II-III-IV, indicating different levels of motor recovery in the upper and lower limbs. The Stroke Impairment Assessment Set (SIAS) was also employed to evaluate the motor function of the paralyzed side, with scores of 1-1C/3-3-3. When interpreted together, both the BRS and SIAS assessments consistently indicated that the patient's upper limb and hand were severely impaired, with limited shoulder movement preventing the hand from reaching the nipple level and the hand showing only partial ability to perform isolated movements. In the lower limb, the assessments revealed marked awkwardness in hip, knee, and ankle movements, suggesting moderate motor control difficulties. These parallel findings from both the BRS and SIAS assessments presented a coherent picture of the patient's motor function, with the upper limb and hand exhibiting severe deficits and the lower limb displaying moderate impairment. The range of motion (ROM) presented no significant issues in terms of passive ROM, specifically in the shoulder, elbow, wrist, and fingers. However, sensation in the left upper and lower limbs was diminished. Cognitive function was formally assessed using the Mini-Mental State Examination (MMSE), in which the patient scored 27/30. Based on this score, the patient’s cognitive function was deemed largely intact and did not pose a barrier to rehabilitation.

The Fugl-Meyer Assessment-Upper Extremity (FMA-UE) is a comprehensive tool designed to evaluate motor function in stroke patients, specifically focusing on movement, coordination, and reflex activity in the upper limb. The patient's FMA-UE score was 17/66, broken down as follows: Shoulder/Elbow: 10/36, Wrist: 0/10, Hand: 7/14, and Speed/Coordination: 0/6. These scores indicated severe impairments, with no functional movement in the wrist, minimal movement in the hand, and significant limitations in the shoulder and elbow. The Motor Activity Log (MAL) measures the real-world use of the affected limb through two scales: amount of use (AOU) and quality of movement (QOM). The patient's scores of AOU 1.5 and QOM 1.0 indicated that the affected upper limb was rarely used in daily activities, and when it was used, the quality of movement remained poor. These results highlighted the challenges the patient faced in incorporating the affected limb into functional tasks, underscoring the need for interventions that would enhance both movement quality and frequency of use in everyday life.

The patient underwent a combination therapy program that integrated mirror therapy with CCFES alongside rehabilitation. The protocol was structured as follows:

Combination therapy program

1. Mirror Therapy Setup

• The patient was seated in a comfortable position with a mirror placed vertically between the affected and unaffected upper limbs. This setup allowed the patient to visually perceive the movements of the unaffected wrist as if they were being executed by the affected wrist, promoting motor imagery and neural reorganization.

2. CCFES Device Utilization

• The CCFES system employed the IVES®︎ low-frequency therapy device (Ozee Giken Co., Ltd., Tokyo, Japan), which integrates myoelectric-evoked electrical stimulation.

• A motion sensor was attached to the unaffected wrist to detect its extension movements. The detected motion then triggered the stimulation of the affected wrist extensors in real time.

• Self-adhesive electrodes were strategically positioned on the proximal and distal portions of the affected forearm’s wrist extensors to ensure effective stimulation.

• Rectangular biphasic electrical pulses with a pulse width of 50 μs were applied, with the pulse intensity calibrated according to the patient’s tolerance level. The standard frequency was set to a range of 40-80 Hz.

• Wrist extension of the affected side was achieved approximately 10-20 times per minute based on the observed movement of the unaffected wrist.

3. Therapy Schedule

• This combined therapy was administered for 20 minutes per session, five times a week, over four months.

• The stimulation intensity was adjusted based on the patient's tolerance level, comfort, and observed muscle contractions, as the IVES®︎ device does not display absolute current values in milliamperes (mA).

• Key functional assessments were conducted at regular intervals to monitor the patient’s improvement in motor function and to guide any necessary adjustments in therapy.

**Figure 2:**
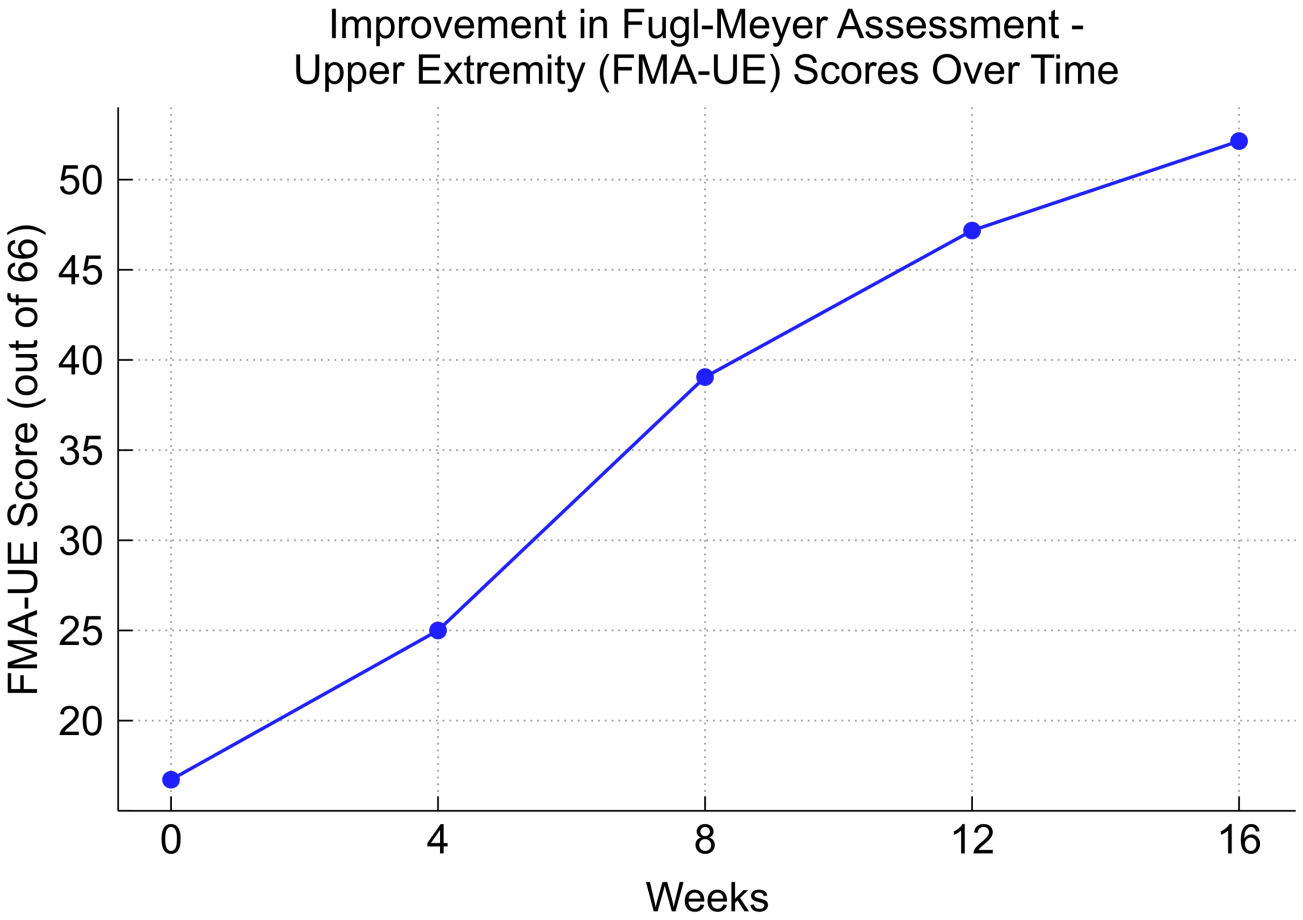
Progression of FMA-UE scores This figure illustrates the changes in FMA-UE scores over 16 weeks. The scores showed consistent improvement from 17/66 at the start of treatment to 52/66 by week 16. This trend indicates a significant recovery of upper extremity function in the subject FMA-UE: Fugl-Meyer Assessment-Upper Extremity

**Figure 3:**
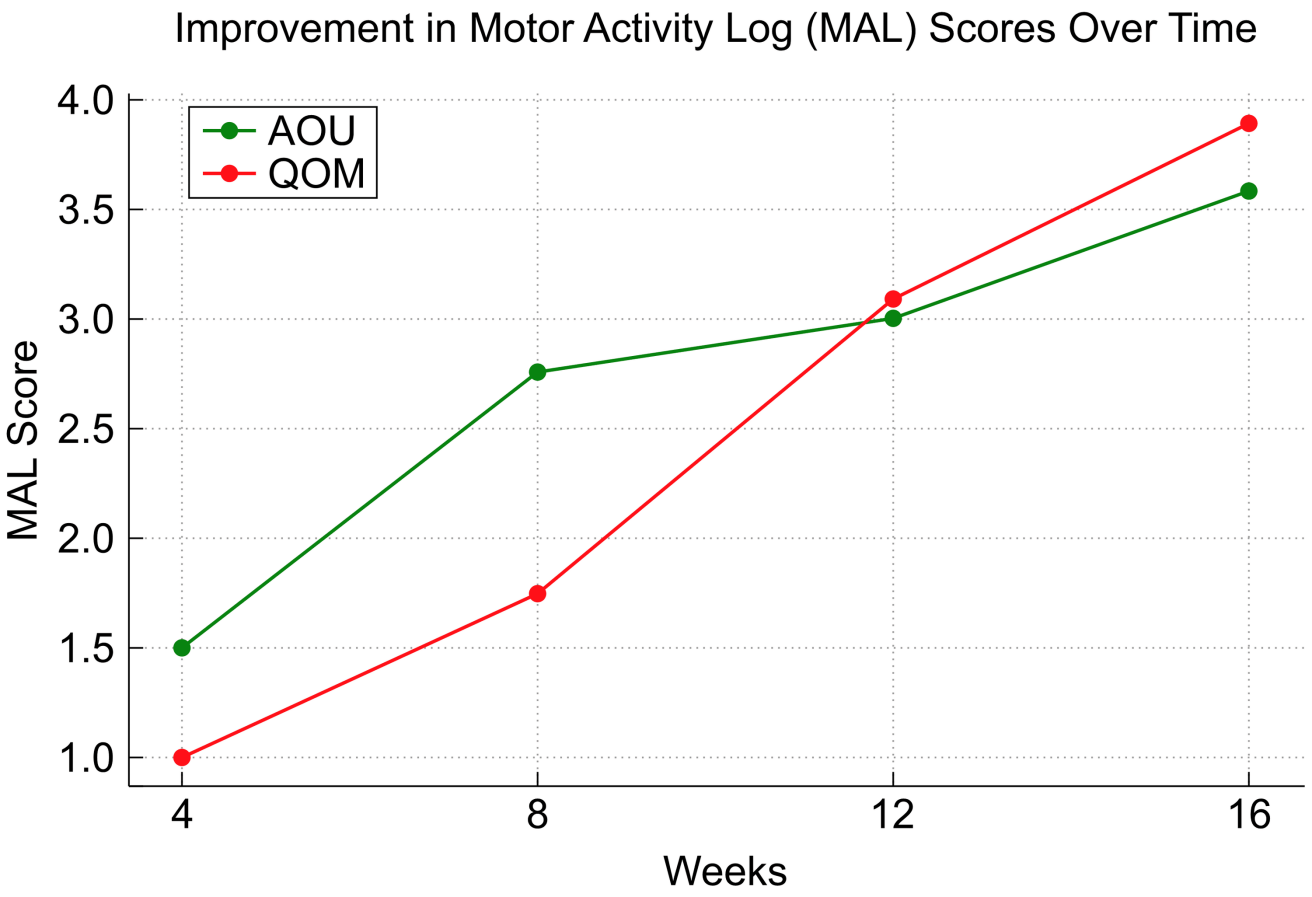
Progression of MAL scores This figure shows changes in the amount of use (AOU) and quality of movement (QOM) scores from the MAL over 16 weeks. The AOU improved from 1.5 to 3.6, while the QOM increased from 1.0 to 3.9, indicating enhanced frequency of use and quality of upper limb movements in daily activities MAL: Motor Activity Log

In this way, adding details of the specific steps and equipment used makes the entire process clearer. At week four, the FMA-UE score was 25/66, with Shoulder/Elbow scores of 14/36, Wrist scores of 4/10, Hand scores of 7/14, and Speed/Coordination of 0/6. The MAL scores were AOU 1.5 and QOM 1.0. By week eight, the FMA-UE score improved to 39/66, with Shoulder/Elbow scores of 24/36, Wrist 6/10, Hand 9/14, and Speed/Coordination 0/6. The MAL scores were AOU 2.75 and QOM 1.75. By week 12, the FMA-UE score further increased to 47/66, with Shoulder/Elbow 26/36, Wrist 7/10, Hand 14/14, and Speed/Coordination 0/6, while the MAL scores were AOU 3.0 and QOM 3.1. Finally, by week 16, the FMA-UE score reached 52/66, with Shoulder/Elbow 28/36, Wrist 8/10, Hand 14/14, and Speed/Coordination 2/6, while the MAL scores were AOU 3.6 and QOM 3.9 (Figures 2, 3).

By week 16, the patient demonstrated significant improvements across multiple measures. The FMA-UE score increased from 17/66 at baseline to 52/66, reflecting substantial gains in motor function across the shoulder, elbow, wrist, and hand. This improvement enabled the patient to perform functional activities with greater ease, such as self-feeding, dressing, and basic grooming tasks, which had been previously challenging. The MAL scores also exhibited notable progress, with AOU improving from 1.5 to 3.6 and QOM from 1.0 to 3.9, indicating that the affected upper limb was not only being used more frequently but also with better control and coordination in daily activities.

## Discussion

This case report highlights the potential efficacy of combining mirror therapy with CCFES in the rehabilitation of severe upper limb motor paralysis following a stroke. The patient demonstrated substantial improvements in motor function, as evidenced by the increase in FMA-UE scores and MAL scores throughout the 16-week intervention period. The notable enhancements observed in this case can be attributed to the synergistic effects of the two therapies. Mirror therapy, which primarily targets visual and cognitive aspects, likely facilitated the activation of the primary motor cortex and enhanced motor imagery, both critical for motor function recovery [7]. Concurrently, CCFES directly stimulates the impaired muscles through electrical stimulation in response to contralateral limb activity, reinforcing neural pathways and fostering bilateral motor coordination [8]. The sensory feedback from CCFES likely complemented the visual input from mirror therapy, creating a comprehensive rehabilitation strategy that engages multiple neural mechanisms simultaneously.

The gradual yet consistent improvement in FMA-UE scores, particularly in the shoulder, elbow, wrist, and hand, suggests that the combined approach effectively restored both gross and fine motor functions. Additionally, the significant progress in the MAL scores [9], reflecting improved quality and quantity of use of the affected limb in daily activities, underscores the functional relevance of the gains achieved through this combined therapy. A key advantage of this combination therapy is its ability to overcome the inherent limitations of each modality [10]. By integrating these approaches, the therapy provides a holistic treatment option that addresses both the neural and muscular aspects of recovery.

However, it is crucial to acknowledge that this report involves a single-patient study, and the results may not be broadly generalizable to all individuals with severe upper limb paralysis. Further research, including larger randomized controlled trials, is needed to validate the efficacy of this combined approach and to explore the underlying neurophysiological mechanisms in greater depth. Additionally, long-term follow-up studies are necessary to determine the sustainability of the observed improvements and assess the potential for achieving functional independence.

## Conclusions

Based on our findings, the combination of mirror therapy and CCFES appears to be a promising strategy for enhancing motor recovery in patients with severe upper limb paralysis following stroke. The significant improvements in motor function and participation in daily activities observed in this case suggest that this approach may offer a valuable addition to rehabilitation toolkits. Future research should focus on optimizing the parameters of this combined therapy and exploring its applicability across diverse patient populations and stages of recovery.
